# Molecular Phylogeny and Zoogeography of the *Capoeta damascina* Species Complex (Pisces: Teleostei: Cyprinidae)

**DOI:** 10.1371/journal.pone.0156434

**Published:** 2016-06-16

**Authors:** Nisreen Alwan, Hamid-Reza Esmaeili, Friedhelm Krupp

**Affiliations:** 1 Senckenberg Research Institute and Museum of Nature, Frankfurt 60325, Germany; 2 Modern University for Business and Science, School of Health Sciences, P.O. Box 113–7501, Beirut, Lebanon; 3 Shiraz University, Department of Biology, College of Sciences, Shiraz 71454, Iran; 4 Qatar Natural History Museum, Qatar Museums Authority, P.O. Box 2777, Doha, Qatar; State Natural History Museum, GERMANY

## Abstract

*Capoeta damascina* was earlier considered by many authors as one of the most common freshwater fish species found throughout the Levant, Mesopotamia, Turkey, and Iran. However, owing to a high variation in morphological characters among and within its various populations, 17 nominal species were described, several of which were regarded as valid by subsequent revising authors. *Capoeta damascina* proved to be a complex of closely related species, which had been poorly studied. The current study aims at defining *C*. *damascina* and the *C*. *damascina* species complex. It investigates phylogenetic relationships among the various members of the *C*. *damascina* complex, based on mitochondrial and nuclear DNA sequences. Phylogenetic relationships were projected against paleogeographical events to interpret the geographic distribution of the taxa under consideration in relation to the area’s geological history. Samples were obtained from throughout the geographic range and were subjected to genetic analyses, using two molecular markers targeting the mitochondrial cytochrome oxidase I (n = 103) and the two adjacent divergence regions (D1-D2) of the nuclear 28S rRNA genes (n = 65). Six closely related species were recognized within the *C*. *damascina* complex, constituting two main lineages: A western lineage represented by *C*. *caelestis*, *C*. *damascina*, and *C*. *umbla* and an eastern lineage represented by *C*. *buhsei*, *C*. *coadi*, and *C*. *saadii*. The results indicate that speciation of these taxa is rather a recent event. Dispersal occurred during the Pleistocene, resulting in present-day distribution patterns. A coherent picture of the phylogenetic relationships and evolutionary history of the *C*. *damascina* species complex is drawn, explaining the current patterns of distribution as a result of paleogeographic events and ecological adaptations.

## Introduction

**Fig 1:**
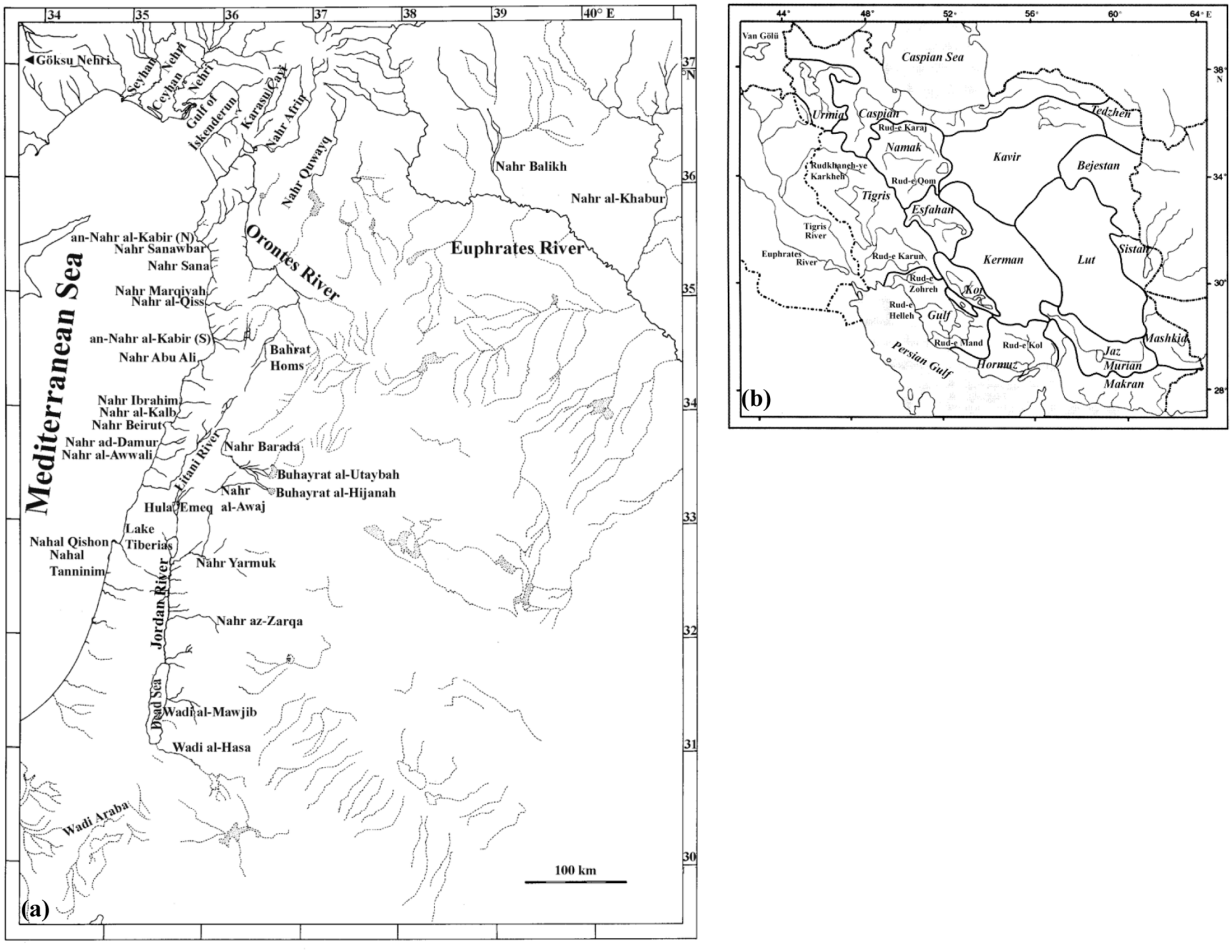
Drainage basins in: (a) Levant (b) Iran (the Maharlu basin lies between the Rud-e Kor and the Gulf basins). Drawing of Fig 1(b) reprinted from [9] under a CC BY license, with permission from author with modifications.

The tectonic events, which started in the Middle East during the Upper Miocene, played a major role in shaping its geomorphological features and had a considerable influence on its fluviatile catchments basins [1–3; Fig 1]. During the Miocene and for much of the Pliocene, the major Levantine river systems (Orontes, Litani, and Jordan) drained to the Euphrates [4–6]. Nahr Quwayq, which was connected to the central course of the Orontes, also drained into the Euphrates [4]. The Yizre’el Valley depression in Palestine and Israel, which was formed during the Upper Miocene, drained the confluence of the Litani River and Jordan River into the Mediterranean during the Pliocene [2]. At that time, the Damascus and Palmyra basins served as an intermediate link between Euphrates and the Jordan-Litani system [5,7]. Connections between the western affluents of the Euphrates River and upper courses of the Ceyhan Nehri also existed during the Pliocene and probably continued during the Pleistocene [8]. However, these fluvial connections did not last.

The uplifting of the southeastern Syrian highlands during the Upper Pliocene ended the connection between the Damascus basin and the Euphrates, while maintaining that between the Damascus basin and the Jordan-Litani system [8]. This latter connection was broken off by the basaltic eruptions of the Hauran and Gabal (Mountain) ad-Duruz during the Upper Pleistocene [8,10]. The uplifting of Gabal az-Zawiyah, caused by the subsidence of the al-Ghab Valley during the Lower Pleistocene, cut the connections between the Proto-Orontes and the Euphrates. At that time, the present Orontes River consisted of three unconnected courses, which used to discharge temporarily into the Mediterranean via coastal rivers [4,6,7,11]. The basaltic extrusions, which erupted during the Quaternary, separated the Orontes from the coastal rivers [6,12]. The confluence of the three formerly separated segments of the Orontes occurred around 6,000 years ago, caused by the effect of retrogressive erosions [4,6,11,13].

The uplifting of the Metulla-Marj Uyun block during the Pleistocene (ca. 2 Ma BP) separated the Litani from the Jordan River. At that time, the downwarping of the Jordan Valley caused the Jordan River to flow in a south-eastward direction into the Jordan Valley [2]. The contact between the Quwayq and the Euphrates was lost very recently attributed to a greater extent to aridity [14]. In addition to these tectonic events, the global sea level dropped by at least 100 m during the Pleistocene glacials (1.82 Ma-11 ka BP), resulting in direct connections among the Levantine coastal rivers by eustatic regressions [7,8,15,16]. Towards the east, the Persian Gulf dried up completely and a river valley conveyed the waters of Mesopotamia to the Gulf of Oman [15,17]. Only some 17,000 years ago, the sea began to rise again reaching its present level some 5,000 years ago, resulting in the separation of these fluvial connections [17].

The paleogeography of the Middle East and the history of its hydrographic systems described above are reflected in the distribution patterns of freshwater fishes in the region. The cyprinid fish *Capoeta damascina* (Valenciennes in Cuvier and Valenciennes, 1842) [18] was earlier considered by many authors as one of the most common freshwater fishes, occurring in a wide range of isolated water bodies in the Levant, Mesopotamia, Turkey, and Iran [7,19–25]. By the nature of its ecology and distribution, this species represents a suitable model to illustrate relationships among geographical areas. Owing to the high variation in morphological characters among and within its various populations, 17 nominal species were described. Several of these species such as *C*. *umbla* (Heckel, 1843) [26] from the Tigris-Euphrates River system, *C*. *saadii* (Heckel, 1849) [27] from the Rud-e Kor (Kor basin), Mand (Persian Gulf basin) and Kol drainages (Hormuz basin), *C*. *buhsei* Kessler, 1877 [28] from Daryacheh-ye Namak (Namak basin) and Kavir basin, *C*. *angorae* (Hankó, 1924) [29] from Seyhan and Ceyhan Nehri drainages, and *C*. *kosswigi* Karaman, 1969 [30] from Van Gölü basin were regarded by some as synonyms of *C*. *damascina* while others regarded them as distinct species [20,23,30–33]. In 2006, the authors [34], without examining any specimens of *C*. *damascina*, restricted its distribution to Syria, Lebanon, and Palestine/Israel. According to them, *C*. *angorae* from the Seyhan and Ceyhan Nehri drainages, *C*. *kosswigi* from Van Gölü basin, and *C*. *umbla* from the Tigris-Euphrates River system were distinct species. In an attempt to genetically classify the species within the genus *Capoeta* from Turkey, the author [35], based on genetic data using the 16S rDNA marker, suggested the conspecificity of *C*. *c*. *umbla* and *C*. *c*. *kosswigi* with *C*. *trutta* (Heckel, 1843) [26]. He stated that *C*. *damascina* and *C*. *barroisi* Lortet in Barrois, 1894 [36] are branched together forming a sister group to *C*. *angorae* and that the former two may be considered subspecies. However, he noted that the application of other genes can help in clarifying these issues. His study also indicated the presence of a new species from Göksu Nehri drainage, which was later described by [37]as *C*. *caelestis*. According to the latter authors, *C*. *angorae* and *C*. *caelestis* belong to a group of superficially similar, almost plain brown, slightly compressed species with narrow lips (*C*. *bergamae* Karaman, 1969 [30]; *C*. *damascina*; *C*. *kosswigi*, and *C*. *umbla*). In an another attempt to understand the inner phylogeny of the genus *Capoeta* using the complete cytochrome *b* gene, the authors [38]considered *C*. *angorae*, *C*. *buhsei*, *C*. *caelestis*, *C*. *damascina*, *C*. *kosswigi*, and *C*. *saadii* as valid species, which are part of an Anatolian-Iranian group occupying the drainages of southeastern Turkey, the Tigris-Euphrates River system, the Iranian inland basins and small rivers draining into the Persian Gulf and Sea of Oman. The Anatolian species (*C*. *angorae*, *C*. *caelestis*, *C*. *damascina*, and *C*. *kosswigi*) form a sister group to their Iranian congeners (*C*. *buhsei* and *C*. *saadii*). The latter authors’ attempt to study the aforementioned species, which are part of what will be referred to in this paper as the “*C*. *damascina* species complex”, remains premature as they studied them very briefly being outside the scope of their investigation. The *C*. *damascina* species complex, as may be derived from the references cited above, includes the following species: *C*. *angorae*, *C*. *buhsei*, *C*. *caelestis*, *C*. *damascina*, *C*. *kosswigi*, *C*. *saadii*, and *C*. *umbla*.

The current study aims at defining *C*. *damascina* and the *C*. *damascina* species complex. It investigates phylogenetic relationships among the various members of the *C*. *damascina* complex and among the populations within each species based on mitochondrial and nuclear DNA sequences and assesses the degree of genetic variation among them. Phylogenetic relationships are subsequently projected against paleogeographic events, interpreting the species’ current geographic distribution patterns and explaining the *C*. *damascina* species complex as a result of recent diversification.

## Materials and Methods

### 2.1. Sample collection

A total of 72 samples of the *C*. *damascina* species complex were collected from 53 localities representative of the entire distribution area using electric fishing gear (EFGI 650; Jürgen Bretschneider Spezialelektronik, Germany), cast and dip nets, and hook and lines. Samples were fixed in 96% ethanol (except for SMF 17353 and AUBM OS3682, which were preserved in 70 and 95% ethanol respectively). They were deposited in the Senckenberg Research Institute and Museum of Nature, Frankfurt, Germany (SMF). Some specimens from Lebanon, Turkey, and Iran were obtained as loans from the American University of Beirut (Natural History) Museum, Beirut, Lebanon (AUBM), Collection of the Biology Department of Shiraz University, Shiraz, Iran (CBSU) and the private collection of Dr. Jörg Freyhof, Berlin, Germany (FSJF: Fischsammlung J. Freyhof). In order to study their phylogenetic relationships with the *C*. *damascina* species complex, samples of other *Capoeta* species (n = 32) such as *C*. *aculeata* (Valenciennes in Cuv. and Val., 1844) [39], *C*. *barroisi*, *C*. *erhani* Turan, Kottelat and Ekmekçi, 2008 [40], *C*. *mandica* Bianco and Banarescu, 1982 [31], *C*. *mauricii* Küçük, Turan, Şahin and Gülle, 2009 [41], *C*. *pestai* (Pietschmann, 1933) [42], *C*. *trutta*, and *C*. *turani* Özuluğ and Freyhof, 2008 [43] were included (deposited in SMF or obtained as loans from FSJF). All samples are listed in Table 1.

**Table 1:** Material used in this study. Accession numbers of COI sequences are written in bold and those of LSU sequences are written in italics.

GenBank Accession No.	Collection No.	Species	Locality	Coordinates	Author	Remarks
**AB238965.1**	-	*Barbus barbus*	Rabnitz,Danube River, Lutzmansburg, Austria	-	[44]	-
-*AF133089*.*2*	-	*Cyprinus carpio*	-	-	[45]	-
-*EF417164*.*1*	-	*Barbus barbus*	-	-	[46]	-
**X61010.1**	-	*Cyprinus carpio*	-	-	[47]	-
**KT385667** *KU948089*	AUBM OS3682	*Capoeta damascina*	Ammiq marsh, Lebanon	33° 43.913' N 35° 47.083' E	This study	Fin clip in 95% alcohol; specimen in 70% alcohol
**KT633581** *KU948088*	AUBM OS3720	*Capoeta damascina*	Nahr al-Kalb estuary, Lebanon	33° 57.303' N 35° 36.005' E	This study	-
**KT633582** *KU948090*	AUBM OS3721	*Capoeta damascina*	Tayr Felsbeh, Lebanon	33° 19.147' N 35° 20.667' E	This study	-
**KT633583** *KU948091*	AUBM OS3724	*Capoeta damascina*	Al-Hasbani, next to Al-Hasbani spring, Lebanon	33° 24.524' N 35° 40.293' E	This study	-
**KT633584**	CBSU uncatalogued (# 1)	*Capoeta saadii*	Kuhmareh Sorkhi, Gulf basin, Iran	-	This study	Fin clip
**KT633585**	CBSU uncatalogued (# 2)	*Capoeta saadii*	Kuhmareh Sorkhi, Gulf basin, Iran	-	This study	Fin clip
**KT633586**	CBSU uncatalogued (# 11)	*Capoeta umbla*	Rud-e Garan, Marivan, Kurdestan, Tigris-Euphrates River system, Iran	-	This study	Fin clip
-*KU948092*	CBSU uncatalogued (# 21)	*Capoeta saadii*	Janatshahr, Fork road, Darab, Hormuz basin, Iran	-	This study	Fin clip
**KT633587**	FSJF 7	*Capoeta coadi*	Rud-e Sangan (Sangan stream) at Sangan, Tigris-Euphrates River system, Iran	31° 15.692' N 51° 17.150' E	This study	Fin clip from FSJF 2213
**KT633588**	FSJF 7	*Capoeta coadi*	Rud-e Sangan (Sangan stream) at Sangan, Tigris-Euphrates River system, Iran	31° 15.692' N 51° 17.150' E	This study	Fin clip from FSJF 2213
**KT633589** *KU948093*	FSJF 7	*Capoeta coadi*	Rud-e Sangan (Sangan stream) at Sangan, Tigris-Euphrates River system, Iran	31° 15.692' N 51° 17.150' E	This study	Fin clip from FSJF 2213
**KT633590** *KU948094*	FSJF 7	*Capoeta coadi*	Rud-e Sangan (Sangan stream) at Sangan, Tigris-Euphrates River system, Iran	31° 15.692' N 51° 17.150' E	This study	Fin clip from FSJF 2213
**KT633591** *KU948095*	FSJF 10	*Capoeta buhsei*	Taghra Rud between Ja’fari and Dolatabad, Daryacheh-ye Namak basin, Iran	34° 42.954' N 50° 27.286' E	This study	Fin clip from FSJF 2206, identified by J. Freyhof
**KT633592**	FSJF 15	*Capoeta saadii*	Golabii spring, 35 km north of Darab, Hormuz basin, Iran	28° 47.255' N 54° 22.321' E	This study	Fin clip from FSJF 2242
**KT633593**	FSJF 17	*Capoeta aculeata*	Taghra Rud between Ja’fari and Dolatabad, Daryacheh-ye Namak basin, Iran	34° 42.954' N 50° 27.286' E	This study	Fin clip from FSJF 2205
**KT633594** *KU948096*	FSJF 18	*Capoeta saadii*	Pirbanoo spring about 10 km south of Shiraz, Daryacheh-ye Maharlu basin, Iran	29° 31.135' N 52° 27.933' E	This study	Fin clip from FSJF 2251, identified by J. Freyhof
**KT633595** *KU948097*	FSJF 22	*Capoeta saadii*	Rud-e Kor about 73 km north of Shiraz, Fars, Rud-e Kor basin, Iran	30° 11.618' N 52° 27.945' E	This study	Fin clip from FSJF 2250
**KT633596** *KU948098*	FSJF 31	*Capoeta mauricii*	Sarıöz Deresi at İsaköy about 4 km south of Sariköy, Turkey	37° 44.908' N 31° 46.818' E	This study	Fin clip from FSJF 1950, identified by J. Freyhof
**KT633597** *KU948099*	FSJF 284	*Capoeta caelestis*	Göksu Nehri at Göksu, below Göksu power station, Turkey	37° 02.740' N 32° 44.562' E	This study	Fin clip from FSJF 2304, identified by J. Freyhof
**KT633598** *KU948100*	FSJF 284	*Capoeta caelestis*	Göksu Nehri at Göksu, below Göksu power station, Turkey	37° 02.740' N 32° 44.562' E	This study	Fin clip from FSJF 2304, identified by J. Freyhof
**KT633599** *KU948101*	FSJF 292	*Capoeta damascina*	Arsuz Nehri (Arsuz stream), east of Arsuz, Turkey	36° 23.950' N 35° 53.158' E	This study	Fin clip from FSJF 2341
**KT633600** *KU948102*	FSJF 299	*Capoeta damascina*	Nehir Yıldırım at Serinyol, Turkey	36° 21.971' N 36° 10.868' E	This study	Fin clip from FSJF 2436, identified by J. Freyhof
**KT633601**	FSJF 353	*Capoeta turani*	Çatkıt Suyu south of Salbaş, the lower part of Pozantı Nehir, Turkey	37° 05.767' N 35° 07.019' E	This study	Fin clip from FSJF 2436, identified by J. Freyhof
**KU893273**	FSJF 353	*Capoeta turani*	Çatkıt Suyu south of Salbaş, the lower part of Pozantı Nehir, Turkey	37° 05.767' N 35° 07.019' E	This study	Fin clip from FSJF 2436, identified by J. Freyhof
**KU893274**	FSJF 353	*Capoeta turani*	Çatkıt Suyu south of Salbaş, the lower part of Pozantı Nehir, Turkey	37° 05.767' N 35° 07.019' E	This study	Fin clip from FSJF 2436, identified by J. Freyhof
**KU893275**	FSJF 353	*Capoeta turani*	Çatkıt Suyu south of Salbaş, the lower part of Pozantı Nehir, Turkey	37° 05.767' N 35° 07.019' E	This study	Fin clip from FSJF 2356, identified by J. Freyhof
**KU892580** *KU948103*	FSJF 355	*Capoeta damascina*	İncesu spring at Hassa, Turkey	36° 47.593' N 36° 30.824' E	This study	Fin clip from FSJF 2275, identified by J. Freyhof
**KU892581** *KU948104*	FSJF 376	*Capoeta damascina*	Pozantı Nehir between Ulukışla and Pozantı, about 1 km east of Çiftehan, Turkey	37° 30.429' N 34° 47.422' E	This study	Fin clip from FSJF 2367
**KU892582** *KU948105*	FSJF 897	*Capoeta damascina*	Upper Göksu Nehri, 5 km northeast of Gölbaşı, Turkey	37° 50.217' N 37° 41.088' E	This study	Fin clip from FSJF 2633
**KU892583** *KU948106*	FSJF 904	*Capoeta damascina*	Affluent canal below Cipköy damlake at picnic area, Turkey	38° 40.753' N 39° 03.962' E	This study	Fin clip from FSJF 2494
**KU892584**	FSJF 919	*Capoeta trutta*	Nehir Çakal, 13 km west of Adıyaman, tributary to Atatürk damlake, Turkey	37° 43.342' N 38° 09.920' E	This study	Fin clip from FSJF 2589, identified by J. Freyhof
**KU892585** *KU948107*	FSJF 935	*Capoeta damascina*	Nehir Çelik at road south of Gölbaşi, Adiyaman, Turkey	37° 37.433' N 37° 30.206' E	This study	Fin clip from FSJF 2571
**KU892586**	FSJF 936	*Capoeta erhani*	Nehir Çelik at road south of Gölbaşı, Turkey	37° 37.433' N 37° 30.206' E	This study	Fin clip; specimen identified by J. Freyhof
**KU892587**	FSJF 936	*Capoeta erhani*	Nehir Çelik at road south of Gölbaşı, Turkey	37° 37.433' N 37° 30.206' E	This study	Fin clip; specimen identified by J. Freyhof
**KU899112** *KU948108*.	FSJF 954	*Capoeta damascina*	Yenice İrmağı (Zamantı stream), south of Aşağıbeyçayırı, south of Pınarbaşı, Turkey	38° 39.354' N 36° 26.910' E	This study	Fin clip from FSJF 2540
**KU899113** *KU948109*	FSJF 1114	*Capoeta pestai*	Çayköy Deresi above Kemerköprü water regulator, southeast of Eğirdir, Turkey	37° 50.253' N 30° 54.046' E	This study	Fin clip from FSJF 2515, identified by J. Freyhof
**KU899114**	FSJF 1308	*Capoeta turani*	Çatkıt Suyu south of Salbaş, the lower part of Pozantı Nehir, Turkey	37° 06.155' N 35° 06.572' E	This study	Fin clip; specimen identified by J. Freyhof
**KU899115**	FSJF 1313	*Capoeta barroisi*	Tahtaköprü east of Islahiye, Turkey	36° 59.185' N 36° 42.276' E	This study	Fin clip; specimen identified by J. Freyhof
**KU899116**	FSJF 1415	*Capoeta trutta*	Nehir Kangal under railway bridge at Çetinkaya, Turkey	39° 15.095' N 37° 37.136' E	This study	Fin clip; specimen identified by J. Freyhof
**KU899117** *KU948110*	FSJF 1425	*Capoeta umbla*	Tigris River, 5 km east of Bismil, Turkey	37° 50.314' N 40° 41.620' E	This study	Fin clip; specimen identified by J. Freyhof
**KU899118**	FSJF 1433	*Capoeta trutta*	Tigris River, 5 km west of Hasankeyf, Turkey	37° 43.429' N 41° 21.630' E	This study	Fin clip; specimen, identified by J. Freyhof
**KU899119** *KU948111*	FSJF 1471–1	*Capoeta damascina*	Tributary to Ceyhan Nehri, between Tecirli and Kadirli north of Koçyurdu, Turkey	37° 13.290' N 36° 02.825' E	This study	Fin clip; specimen identified by J. Freyhof
**KU899120** *KU948112*	FSJF 1471–2	*Capoeta damascina*	Tributary to Ceyhan Nehri, between Tecirli and Kadirli north of Koçyurdu, Turkey	37° 13.290' N 36° 02.825' E	This study	Fin clip; specimen identified by J. Freyhof
**KU899121** *KU948113*	FSJF 1494	*Capoeta umbla*	Outflow of Hazar Gölü at Plajköy, Tigris-Euhprates River system, Turkey	38° 30.187' N 39° 30.423' E	This study	-
**KU899122**	SMF 17353	*Capoeta damascina*	An-Nahr al-Kabir (S), Lebanon	34° 40' N 36° 18' E	This study	Specimen in 70% alcohol
**KU899123**	SMF 30733	*Capoeta mandica*	Rudkhaneh-ye Rudbal near Firuzabad, Iran	28° 42.590' N 52° 38.222' E	This study	-
**KU899124**	SMF 30855	*Capoeta mandica*	Qareh Aghaj, Iran	28° 49.978' N 53° 20.005' E	This study	-
**KU899125**	SMF 30856	*Capoeta trutta*	Rud-e Fahlian, Iran	30° 11.143' N 51° 31.247' E	This study	-
**KU899126**	SMF 30858	*Capoeta mandica*	Pol-e Qareh Aghaj, Iran	29° 41.217' N 52° 06.003' E	This study	-
**KU899127** *KU948114*	SMF 30861	*Capoeta saadii*	Small spring 55 km from Shahr-e Babak, Javazm village, Kerman basin, Iran	30° 30.882' N 55° 01.902' E	This study	-
**KU899128**	SMF 30862	*Capoeta trutta*	Rudkhaneh-ye Karkheh near Pol-e Dokhtar, Iran	33° 09.602' N 47° 43.195' E	This study	-
**KU899129**	SMF 30863	*Capoeta trutta*	Rud-e Tang-e Sheeb in Kupan, Iran	30° 19.343' N 51° 14.535' E	This study	-
**KU899130**	SMF 30864	*Capoeta mandica*	Rudkhaneh-ye Rudbal near Firuzabad, Iran	28° 42.590' N, 52° 38.222' E	This study	-
**KU899131** *KU948115*	SMF 30865	*Capoeta coadi*	Tang-e Sorkh, Tigris-Euphrates River system, Iran	30° 27.680' N 51° 44.907' E	This study	-
**KU899132**	SMF 30867	*Capoeta aculeata*	Tang-e Sorkh, Tigris-Euphrates River system, Iran	30° 27.680' N 51° 44.907' E	This study	-
**KU899133**	SMF 30869	*Capoeta mandica*	Pol-e Qareh Aghaj, Iran	29° 41.217' N 52° 06.003' E	This study	-
**KU925891**	SMF 30870	*Capoeta aculeata*	Rud-e Tang-e Tizab, Sepidan, Fars, Tigris-Euphrates River system	30° 23.470' N 51° 46.710' E	This study	-
**KU925892** *KU948116*	SMF 30871	*Capoeta coadi*	Tang-e Sorkh, Tigris-Euphrates River system, Iran	30° 27.680' N 51° 44.907' E	This study	-
**KU925893** *KU948117*	SMF 30872	*Capoeta coadi*	Rud-e Tang-e Tizab, Sepidan, Fars, Tigris-Euphrates River system, Iran	30° 23.470' N 51° 46.710' E	This study	-
**KU925894** *KU948118*	SMF 30981	*Capoeta damascina*	Nahr Beirut at Qanatir Zubaydah, al-Hazimiyah, Lebanon	33° 50.781' N 35° 30.503' E	This study	-
**KU925895** *KU948119*	SMF 30982	*Capoeta damascina*	Nahr Beirut at Qanatir Zubaydah, al-Hazimiyah, Lebanon	33° 50.781' N 35° 30.503' E	This study	-
**KU925896** *KU948120*	SMF 30983	*Capoeta damascina*	Nahr Abu Ali at Sir‛il (Sera’al), Lebanon	34° 16.982' N 35° 55.729' E	This study	-
**KU934301**	SMF 30984	*Capoeta damascina*	Nahr Abu Ali at Sir‛il (Sera’al), Lebanon	34° 16.982' N 35° 55.729' E	This study	-
**KU948047** *KU948121*	SMF 30985	*Capoeta damascina*	Nahr Bisri leading to Nahr al-Awwali, Lebanon	33° 34.823' N 35° 32.126' E	This study	-
**KU948048** *KU948122*	SMF 30987	*Capoeta damascina*	Nahr Antelias at Antelias, Lebanon	33° 54.748' N 35° 35.760' E	This study	-
**KU948049** *KU948123*	SMF 30990	*Capoeta damascina*	Nahr al-Qasimiyah, Lebanon	33° 19.207' N 35° 17.291' E	This study	-
**KU948050** *KU948124*	SMF 30991	*Capoeta damascina*	Nahr al-Kalb at magharat Jeita (J’ita/Jeita Grotto) below the cave, Lebanon	33° 56.340' N 35° 39.092' E	This study	-
**KU948051** *KU948125*	SMF 30992	*Capoeta damascina*	Nahr al-Awwali below the bridge, Lebanon	33° 35.288' N 35° 23.630' E	This study	-
**KU948052** *KU948126*	SMF 30994	*Capoeta damascina*	Nahr Kafr Matta at Jisr al-Kadi, Lebanon	33° 43.297' N 35° 33.474' E	This study	-
**KU948053** *KU948127*	SMF 30995	*Capoeta damascina*	Nahr Kafr Matta at Jisr al-Kadi, Lebanon	33° 43.297' N 35° 33.474' E	This study	-
**KU948054**	SMF 30997	*Capoeta aculeata*	River at Band-e Amir, Iran	29° 46.500' N 52° 50.612' E	This study	Fin clip
**KU948055**	SMF 30998	*Capoeta aculeata*	Zayandeh Rud in Esfahan, Esfahan basin, Iran	32° 38.327' N 51° 36.738' E	This study	Fin clip; whole specimen present at the University of Tehran
**KU948056**	SMF 30999	*Capoeta aculeata*	Rud-e Hadi between Zagheh and Polehoru, Tigris-Euphrates River system, Iran	33° 31.133' N 48° 46.340' E	This study	Fin clip
**KU948057**	SMF 31000	*Capoeta aculeata*	Rud-e Qom in Qom, Daryacheh-ye Namak basin, Iran	34° 22.623' N 50° 36.105' E	This study	-
**KU948058**	SMF 31001	*Capoeta mandica*	Rudkhaneh-ye Rudbal near Firuzabad, Iran	28° 42.590' N 52° 38.222' E	This study	-
**KU948059**	SMF 31002	*Capoeta aculeata*	Rud-e Qom in Qom, Daryacheh-ye Namak basin, Iran	34° 22.623' N 50° 36.105' E	This study	Fin clip
**KU948060** *KU948128*	SMF 31003	*Capoeta buhsei*	Qareh Su (Qara Chai) in Tureh, Daryacheh-ye Namak basin, Iran	34° 02.118' N 49° 16.970' E	This study	Fin clip
**KU948061** *KU948129*	SMF 31004	*Capoeta buhsei*	Pol-e Doab, Arak-Markazi, Daryacheh-ye Namak basin, Iran	34° 02.607' N 49° 21.157' E	This study	-
**KU948062** *KU948130*	SMF 31005	*Capoeta saadii*	Rudkhaneh-ye Rudbal, Fars, Gulf basin, Iran	28° 42.504' N 52° 36.631' E	This study	-
**KU948063**	SMF 31007	*Capoeta saadii*	Kohmareh Sorkhi, Shiraz, Fars, Gulf basin, Iran	29° 23.728' N 52° 09.650' E	This study	-
**KU948064** *KU948131*	SMF 31008	*Capoeta saadii*	Kohmareh Sorkhi, Shiraz, Fars, Gulf basin, Iran	29° 23.728' N 52° 09.650' E	This study	-
**KU948065** *KU948132*	SMF 31010	*Capoeta saadii*	Sarab spring-stream system, Fars, Rud-e Kor basin, Iran	29° 50.810' N 52° 25.211' E	This study	Fin clip; specimen identified by H. R. Esmaeili
**KU948066** *KU948133*	SMF 31011	*Capoeta damascina*	Nahr Ibrahim at Shwan, Lebanon	34° 04.916' N 35° 47.100' E	This study	-
**KU948067** *KU948134*	SMF 31012	*Capoeta damascina*	Nahr Ibrahim at Shwan, Lebanon	34° 04.916' N 35° 47.100' E	This study	-
**KU948068** *KU948135*	SMF 31028	*Capoeta damascina*	Small stream at Wadi Shuayb, Jordan	31° 56.205' N 35° 40.003' E	This study	-
**KU948069** *KU948136*	SMF 31029	*Capoeta damascina*	Bahrat Homs (Lake Qattinah), Syria	34° 39.722' N 36° 37.10' E	This study	Fin clip
**KU948070** *KU948137*	SMF 31031	*Capoeta damascina*	Bahrat Homs, Syria	34° 39.722' N 36° 37.10' E	This study	Fin clip from FSJF 2705 (SYR08/25)
**KU948071** *KU948138*	SMF 31033	*Capoeta damascina*	Orontes at al-Qusayr village, Syria	34° 30.515' N 36° 32.340' E	This study	-
**KU948072** *KU948139*	SMF 31034	*Capoeta damascina*	An-Nahr al- Kabir (N) at al-Qastal village, Syria	35° 44.267' N 36° 06.235' E	This study	-
**KU948073** *KU948140*	SMF 31036	*Capoeta damascina*	Wadi Hasa, Jordan	30° 59.015' N 35° 40.228' E	This study	-
**KU948074** *KU948141*	SMF 31038	*Capoeta damascina*	Nahr al-Tammasiyyat near al-Maqsufa, Syria	33° 17.611' N 35° 58.240' E	This study	Fin clip
**KU948075** *KU948142*	SMF 31039	*Capoeta damascina*	Bahrat Homs, Syria	34° 39.722' N 36° 37.100' E	This study	Fin clip
**KU948076** *KU948143*	SMF 31040	*Capoeta damascina*	Abu Noah spring, Syria	34° 56.608' N 35° 53.047' E	This study	Fin clip
**KU948077** *KU948144*	SMF 31044	*Capoeta damascina*	An-Nahr al-Kabir (N) at as-Safkun, Syria	35° 39.360' N 35° 59.835' E	This study	-
**KU948078**	SMF 31046	*Capoeta barroisi*	Bahrat Homs, Syria	34° 39.722' N 36° 37.100' E	This study	Fin clip
**KU948079** *KU948145*	SMF 31047	*Capoeta damascina*	Nahr Marqiyah, Syria	35° 01.828' N 35° 54.298' E	This study	-
**KU948080** *KU948146*	SMF 31049	*Capoeta damascina*	Nahr Marqiyah, Syria	35° 01.828' N 35° 54.298' E	This study	-
**KU948081** *KU948147*	SMF 31050	*Capoeta damascina*	Abu Noah headwater/Nahr Azak, Syria	34° 57.617' N 35° 58.545' E	This study	-
**KU948082** *KU948148*	SMF 31054	*Capoeta damascina*	Spring of Nahr Barada/canal near Barada source, Syria	33° 40.518' N 36° 03.330' E	This study	-
**KU948083** *KU948149*	SMF 31056	*Capoeta damascina*	Spring of Nahr Barada/canal near Barada source, Syria	33° 40.518' N 36° 03.330' E	This study	-
**KU948084** *KU948150*	SMF 31059	*Capoeta damascina*	Nahr al-Yarmuk at Wadi Jallayn, Jordan	32° 44.347' N 35° 58.933' E	This study	-
**KU948085** *KU948151*	SMF 31061	*Capoeta damascina*	Wadi al-Mawjib near the dam, Jordan	31° 26.79' N 35° 48.963' E	This study	-
**KU948086**	SMF 31064	*Capoeta trutta*	Euphrates River with no exact locality, Syria	-	This study	Fin clip taken from a specimen found at fish market
**KU948087** *KU948152*	SMF 33094	*Capoeta saadii*	Small spring 55 km from Shahr-e Babak, Javazm village, Kerman basin, Iran	30° 30.882' N 55° 01.902' E	This study	Fin clip

### 2.2. Ethics Statement

This study was carried out in strict accordance with applicable national and international guidelines. The research work in Iran was funded by Shiraz University and by the German Academic Exchange Service (DAAD) and was approved by the Ethics Committee of Biology Department (SU-909789).

Permission to carry out research in Iran, Lebanon, Syria, and Jordan was not required as unregulated animals were collected. Despite this fact, requests for approval were submitted to the Ministry of Environment and Ministry of Agriculture in the aforementioned countries. The Ministries stated that there are no regulations regarding collected animals. Therefore, no specific permissions were required for localities/activities for field work. The field study did not involve endangered or protected species.

The samples from Turkey included in this study were obtained from the private collection of Dr. Jörg Freyhof. Sampling was not conducted by the authors of this paper. Nevertheless, permission of sampling was obtained by the collectors as confirmed upon delivery of samples.

Collection of fishes was performed with all efforts made to minimize suffering.

### 2.3. DNA extraction, PCR amplification, and sequencing

Prior to DNA extraction, about 25 mg of a muscle tissue taken from the region below the base of the dorsal fin or a fin clip sample (n = 104) were cut using sterile razor blades and placed inside sterile Eppendorf tubes. Subsequently, they were washed twice, one hour each time, with 1 ml Phosphate Buffered Saline (PBS) solution (pH 7.2; Biochrom, Germany) to remove the fixative. After the PBS was discarded, total genomic DNA was extracted with the DNeasy Blood and Tissue kit (QIAGEN, Germany) according to manufacturer’s instructions (animal tissues protocol).

The extracted DNA of *Capoeta* samples was amplified, via PCR, using primer pairs of two molecular sequence markers. The first one targets the mitochondrial cytochrome oxidase I (COI) gene and the second addresses the two adjacent divergence regions (D1–D2) of the large subunit (LSU or 28S) ribosomal RNA gene.

A total of 103 DNA samples were amplified using the COI marker and 65 using the LSU. Approximately 655 base pairs (bp) were amplified from the 5' region of the COI gene using the primer pair FishF1 (5'TCAACCAACCACAAAGACATTGGCAC3') and FishR1 (5'TAGACTTCTGGGTGGCCAAAGAATCA3') adapted from[48]. Regarding the LSU gene, the forward primer D1–D2 LSU F (5'ACAAGTACCGTGAGGGAAAGTTG3') was developed by [46]and modified here. The reverse primer D1–D2 LSU R (5'GGCCTTCACCTTCATTGC3') was designed based on the partial LSU sequence of *Barbus barbus* from GenBank (GenBank: EF417164.1; [46]) and tested using the Primer3 software [49]. This primer pair targets an approximately 616 bp fragment of the D1–D2 region of the LSU ribosomal gene.

Standard PCR was performed in a total volume of 25 μl reaction mixture containing 1 μl of each primer (10 pmol/μl), 5 μl of the DNA template (30–50 ng/μl) and 18 μl of sterile double distilled water (ddH_2_O) in 0.2 ml thin-walled PCR tubes enclosing the illustra^™^ puReTaq Ready-To-Go PCR beads (GE Healthcare, USA). The PCR conditions for the FishF1+ FishR1 primer pair were as follows: Initial denaturation at 94°C (1 min), 40 cycles at 94°C (0.5 min), 52°C (1.5 min), 72°C (1 min), and a final extension at 72°C for 10 min. The PCR protocol for the D1–D2 LSU F + D1–D2 LSU R primer pair encompassed an initial denaturation at 94°C (1 min), 40 cycles at 94°C (0.5 min), 55°C (1.5 min), 72°C (1 min), and a final extension at 72°C (10 min). The PCR products were visualized on 1% agarose gel. In some cases and only when using the D1–D2 primers, more than one band were observed on the gel: One at the exact specified size and another, which is either higher or lower than the previous one. This could be evidence for the presence of pseudogenes or for polymorphism, where multiple copies of ribosomal genes are present in the genome retaining more or less identical sequences. In such cases, the PCR products at both bands were sequenced and both sequences were blasted to identify which one was the partial LSU sequence.

The PCR products were purified with the QIAquick Gel Extraction kit (QIAGEN, Germany) following the “QIAquick Gel Extraction Kit protocol using a microcentrifuge”. The purified PCR products were then sequenced according to the protocol of the Big Dye^®^ v3.1 Cycle Sequencing Kit (Applied Biosystems, Germany) and read on an ABI 3730 capillary sequencer (Applied Biosystems, Germany). Sequencing was done with the same primers used in the PCR reactions. In order to control sequence accuracy and to resolve any ambiguous bases, the PCR products were sequenced in both directions. All sequences are deposited in GenBank (Accession numbers: KT385667-633601, KU948089-948152; Table 1).

### 2.4. Phylogenetic analyses

Sequences were proof-read and assembled using the Lasergene SeqMan II software (DNA Star 6 Inc., USA) and were manually checked for inconsistencies. They were aligned using the ClustalW algorithm [50]with default parameters within MEGA4.0.2 software [51] and visually inspected. Sequences were analyzed in PAUP* 4.0b10 [52] in order to determine the number of variable and parsimony-informative sites.

Sequences of *Cyprinus carpio* (COI: CoxI X61010.1, [47]/LSU: AF133089.2, [45]) and *Barbus barbus* (COI: AB238965.1, [44]/LSU: EF417164.1, [46]) obtained from GenBank were also included in the analyses but only that of *C*. *carpio* was used to root the trees. This is because *C*. *carpio* is one of the closest relatives to our ingroup and does neither cluster with members of the genus *Capoeta* nor with the *Luciobarbus* lineage/*Barbus* sensu stricto group, which were shown to display close phylogenetic relationships with each other, based on mitochondrial gene sequences [44,53–54].

Phylogenetic trees from aligned sequences were constructed using Maximum Parsimony (MP) and Bayesian analysis (BA) for both markers. The MP analysis, with heuristic search using the tree bisection and reconnection branch-swapping option, 1,000 bootstrap replicates and five independent search runs per replicate and random addition of sequences, were performed with PAUP* 4.0b10. Samples with the same haplotypes were excluded and are only represented by one sequence. For BA, the best-fit model of molecular evolution was determined with Mr. Modeltest 2.3 [55] in PAUP* 4.0b10 according to the Akaike Information Criterion (AIC). The subsequent analysis was carried out with the most appropriate model using MrBayes 3.1.2 [56] for six million generations with four chains, a sample frequency of 1,000 generations and a burn-in of 1001 in two separate runs. A total of 66 COI and LSU sequences were combined in a total evidence tree to improve the overall resolution among the clades. The total evidence tree was analyzed using MP and BA. The MP analysis was performed as mentioned above. For a Bayesian reconstruction of phylogeny, the analysis was carried out using MrBayes 3.1.2 for five million generations with four chains, a sample frequency of 1,000 generations and a burn-in of 1001 in two separate runs. The data set was divided into two partitions, one for the COI and one for the LSU. The models of evolution for each partition were specified as stated above.

To display the mitochondrial sequence variation underlying the phylogenetic analysis, haplotype networks were constructed for the COI sequences of the *C*. *damascina* species complex using the TCS 1.21 program [57]. The connection limit was set to 10 mutation steps.

## Results

### 3.1. COI

The COI sequences of 581 nucleotides were obtained for each of the 105 specimens (including two sequences from GenBank) after editing and were unambiguously aligned. Among the 581 nucleotide sites, 455 were constant, 126 were variable and 83 were parsimony informative. The nucleotide composition of the COI sequences was **G**-deficient (16.9%) whereas similar frequencies were observed for the other three nucleotides (**A**: 27.1%, **C**: 28.7%, **T**: 27.3%). The Hasegawa-Kishono-Yano model of molecular evolution [58] with invariant sites and gamma distribution (HKY+I+G) was the best-fitting model for the data set using the AIC.

**Fig 2:**
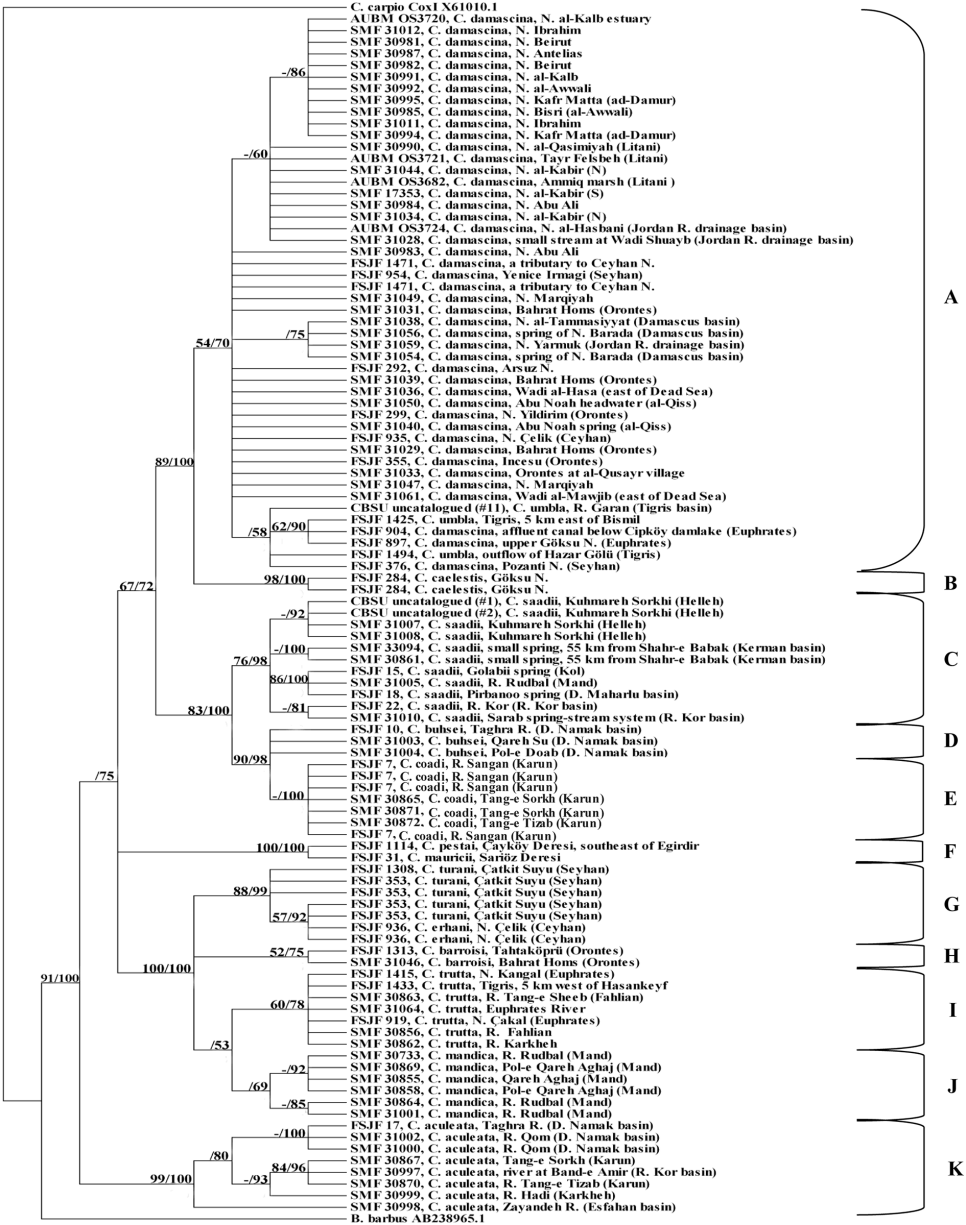
Condensed cladogram obtained from COI sequences using Maximum Parsimony/Bayesian analysis. Numbers above branches refer to bootstrap/posterior probability percentages; only values ≥ 50% are shown. “-” indicates that no bootstrap value was obtained from MP analysis as only a single sequence was included in the analysis.

**Fig 3:**
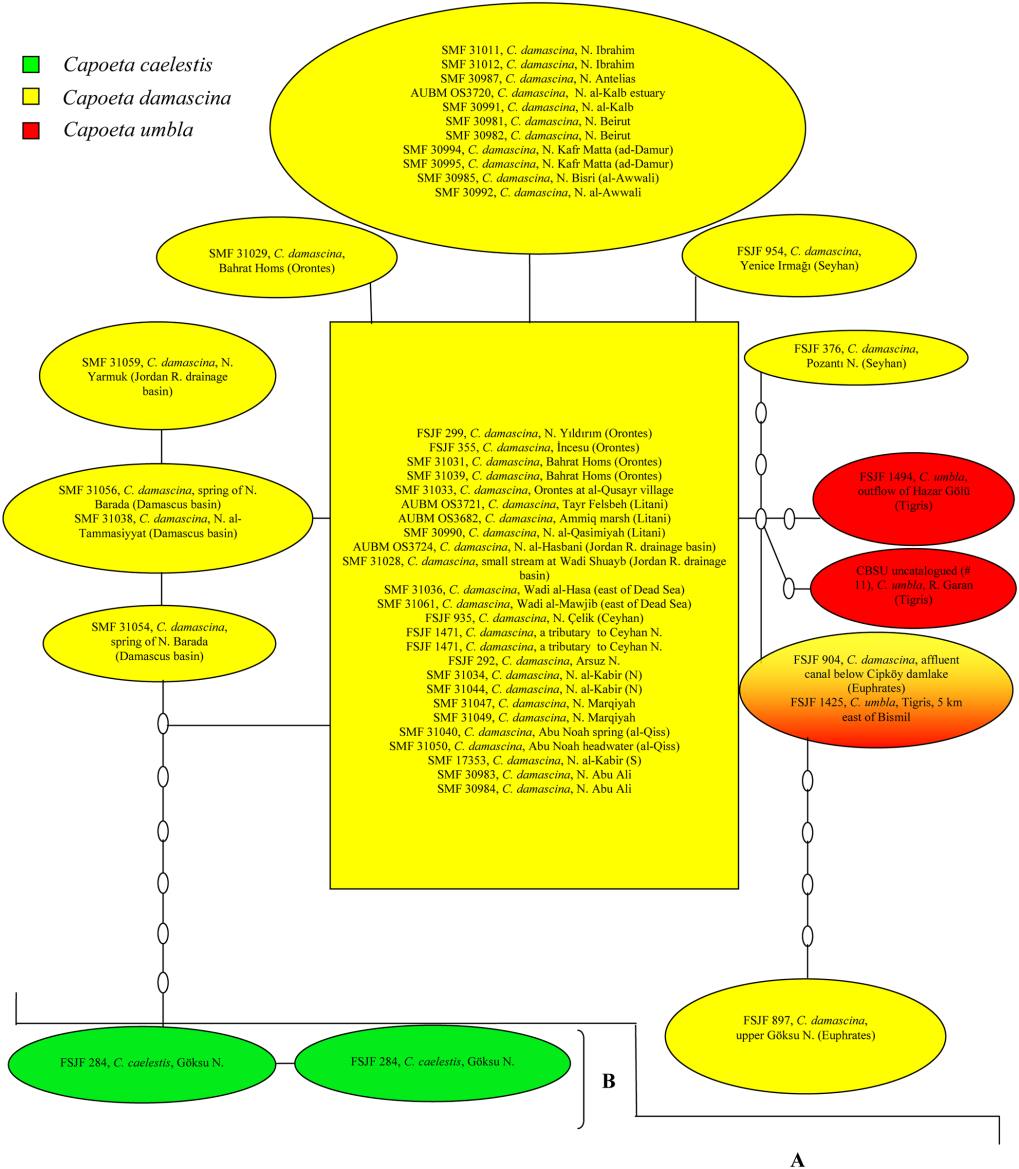
Haplotype network for the *Capoeta caelestis*, *Capoeta damascina*, and *C*. *umbla* COI sequences showing the number of nucleotide differences between haplotypes. Clades labeled A and B correspond to clades A and B in the phylogenetic tree.

**Fig 4:**
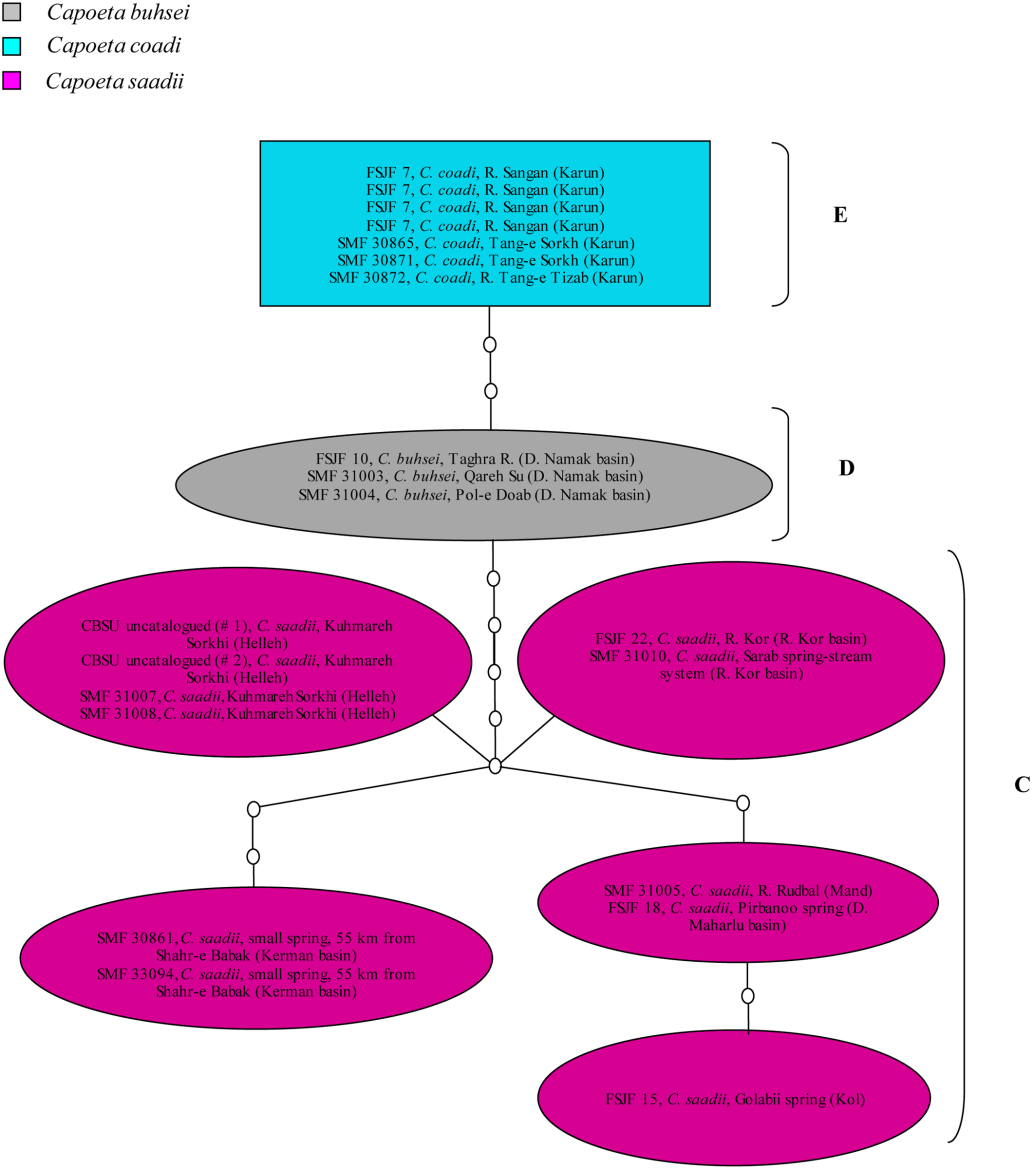
Haplotype network for the *Capoeta buhsei*, *Capoeta coadi*, and *Capoeta saadii* COI sequences. Clades labeled C, D, and E correspond to clades C, D, and E in the phylogenetic tree. These clades are not linked to clades A and B as the number of nucleotide differences exceeds the chosen connection limit (10 mutation steps).

The resulting phylogenetic trees using the MP and the BA methods were congruent. The condensed cladogram (Fig 2) showed that a monophyletic group (A-E) consisting of six closely related species can be recognized within the *C*. *damascina* complex: *C*. *buhsei*, *C*. *caelestis*, *C*. *damascina*, *C*. *saadii*, *C*. *umbla*, and a recently described new species *C*. *coadi* Alwan et al., 2016 [59]. This monophyletic group (A-E) is separate from all remaining species included in this study (bootstrap value = 67%, PP value = 72%). Within this group, two main lineages are identified: A western lineage comprising the fishes from the Levant, Mesopotamia, and parts of southern Turkey (Clade A+B) and an eastern lineage comprising the fishes from Iran (Clade C+D+E) (Fig 2). This is well supported by the haplotype networks (Figs 3 and 4).

In the western lineage, *C*. *caelestis* (clade B, bootstrap value = 98%, PP value = 100%) from Göksu Nehri drainage forms the sister group to the clade, which consists of *C*. *damascina* and *C*. *umbla* (clade A).

Within clade A, *C*. *umbla* is nested within *C*. *damascina* where *C*. *umbla* from the Tigris River system cluster in one group with one sequence of *C*. *damascina* from the Seyhan Nehri drainage (FSJF 376) and two from the Euphrates River system (FSJF 897 and FSJF 904).

Regarding the different *C*. *damascina* populations, the relationships among them are not well resolved though most of the sequences from the coastal rivers of Lebanon tend to cluster with each other, supported by a PP value of 86%. A larger clade with a PP value of 60% contains the remaining sequences from the coastal rivers of Lebanon and four additional ones from the Jordan River drainage basin (two sequences) and from the Syrian coastal river, an-Nahr al-Kabir (N) (two sequences). Similarly, *C*. *damascina* sequences from the Damascus basin tend to cluster together along with one sequence from Nahr Yarmuk in the Jordan River drainage basin (PP value = 75%).

Regarding the eastern lineage which consists of three species (*C*. *buhsei*, *C*. *coadi*. and *C*. *saadii*), it is shown that *C*. *saadii* forms the sister group to *C*. *buhsei* and *C*. *coadi* (clade D+E). *Capoeta buhsei* (clade D) is very closely related to *C*. *coadi*, which together form a well-supported monophyletic group (PP value = 100%).

As shown in Fig 3, most specimens from different *C*. *damascina* populations (clade A) share one of the two most common haplotypes or possess very similar ones. These haplotypes are much more similar to *C*. *umbla* haplotypes (clade A) than to the two *C*. *damascina* haplotypes from the Seyhan Nehri drainage and the Euphrates River system (FSJF 376 and FSJF 897). Interestingly, the two haplotypes obtained for the Seyhan Nehri drainage are very distinct from each other (separated by five mutation steps) and do not form part of the groups that share the two most common haplotypes. *Capoeta umbla* from the Tigris River system (FSJF 1425) shares the same haplotype with *C*. *damascina* from Euphrates (FSJF 904). Although linked to clade A, *C*. *caelestis* (clade B) forms a separate group (seven steps).

Regarding clades C, D, and E (Fig 4), the haplotype network has revealed that *C*. *coadi* is closely related to *C*. *buhsei* (three steps). Interestingly, the *C*. *saadii* haplotypes were quite divergent from the haplotypes of *C*. *buhsei* and *C*. *coadi* (maximum eight steps) and displayed a pattern without an obvious central haplotype. Additionally, the *C*. *saadii* sequences from each separate basin shared the same haplotype, except those from Rud-e Mand drainage and Daryacheh-ye Maharlu basin (two sequences), which clustered together and shared the same haplotype.

### 3.2. LSU

Since the target taxon in this study is the *C*. *damascina* species complex, not all the specimens used in COI analysis were sequenced with the LSU marker. A total of 65 sequences (with a length of 528 sites or positions including nucleotides and gaps) were obtained from *C*. *buhsei*, *C*. *caelestis*, *C*. *coadi*, *C*. *damascina*, *C*. *pestai*, *C*. *saadii*, and *C*. *umbla* individuals. One specimen from the Rud-e Kol drainage (FSJF 15) yielded a very short sequence due to an amplification artifact; therefore, it was replaced by another specimen from the same river drainage but from a different locality (CBSU uncatalogued, # 21). Among the 528 nucleotide sites, 444 were constant, 84 were variable and 44 were parsimony informative. Visual inspection revealed that there was no need for manually improving the alignment. The nucleotide composition of the LSU sequences was as follows: **A**: 15.8%, **C**: 30.8%, **G**: 35.6%, and **T**: 17.8%. The generalized time reversible model [60] with invariant sites (GTR+I) was the best-fitting model of sequence evolution for the data set using the AIC.

**Fig 5:**
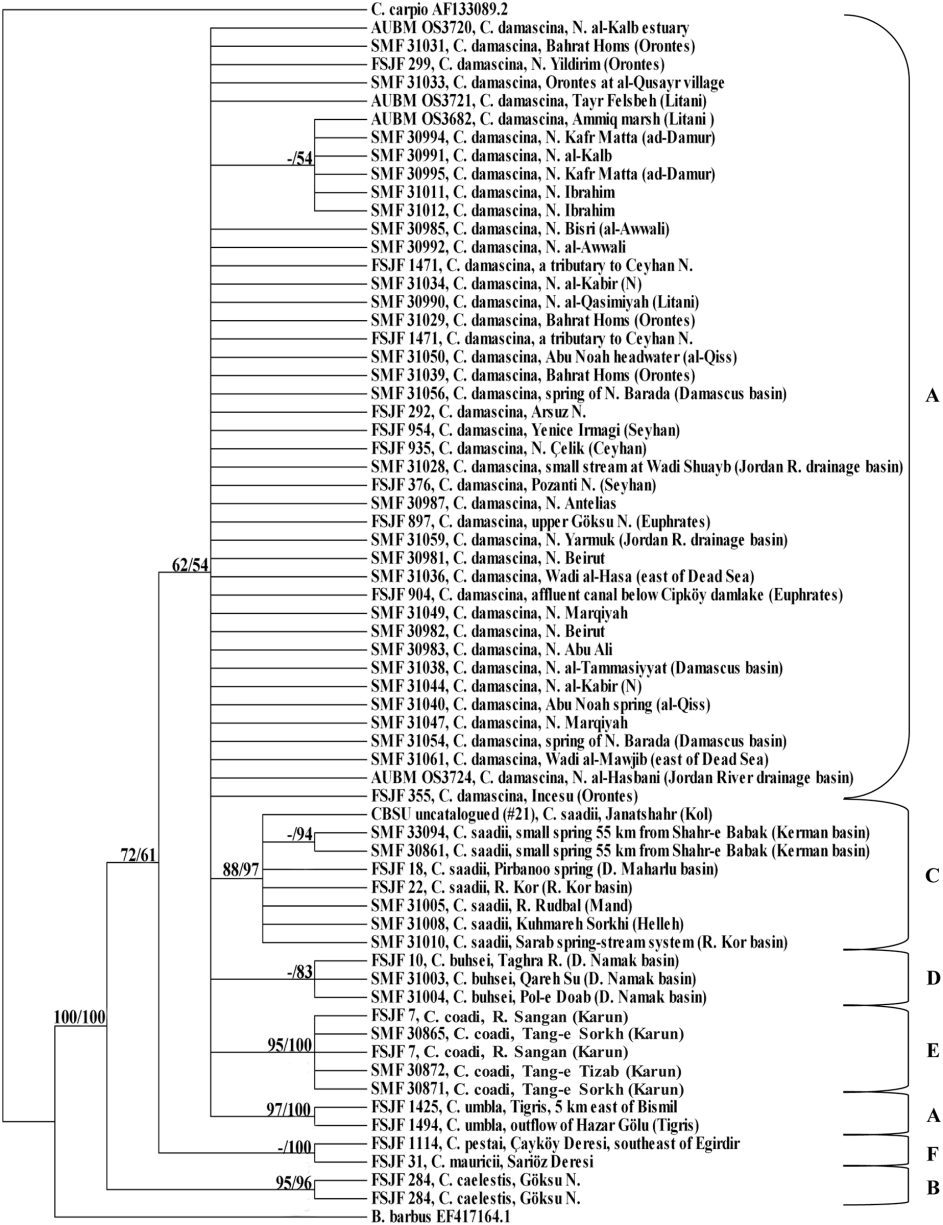
Condensed cladogram obtained from LSU sequences using Maximum Parsimony/Bayesian analysis. Numbers above branches refer to bootstrap/posterior probability percentages; only values ≥ 50% are shown. “-” indicates that no bootstrap value was obtained from MP analysis as only a single sequence was included in the analysis.

The MP and the BA trees show the same topology. The phylogenetic relationships among the different clades are not very well resolved but the tree topology using the LSU marker (Fig 5) supports the monophyly of *C*. *umbla* (clade A), *C*. *caelestis* (clade B), *C*. *saadii* (clade C), *C*. *buhsei* (clade D), *C*. *coadi* (clade E), and *C*. *pestai*/*mauricii* (clade F) with high bootstrap values ranging between 88% and 97% and PP values ranging between 83% and 100%.

Concerning *C*. *damascina* (clade A), the phylogenetic relationships among its individual populations are not well resolved. *Capoeta umbla*, which clustered in one group with few sequences of *C*. *damascina* from the Euphrates River system and the Seyhan Nehri drainage in the previous tree using the COI marker (Fig 2), form a monophyletic group without *C*. *damascina* in the tree using the LSU marker (Fig 5). However, the phylogenetic relationship between *C*. *damascina* and *C*. *umbla* is not resolved. *Capoeta caelestis* (clade B), which formed the sister group to clade A using the COI marker, formed a separate branch, which is basal to all the other *Capoeta* clades using the LSU marker but is not very strongly supported (clade A+C+D+E: bootstrap value = 62%, PP value = 54%; clade A+C+D+E+F: bootstrap value = 72%, PP value = 61%).

### 3.3. COI+LSU

**Fig 6:**
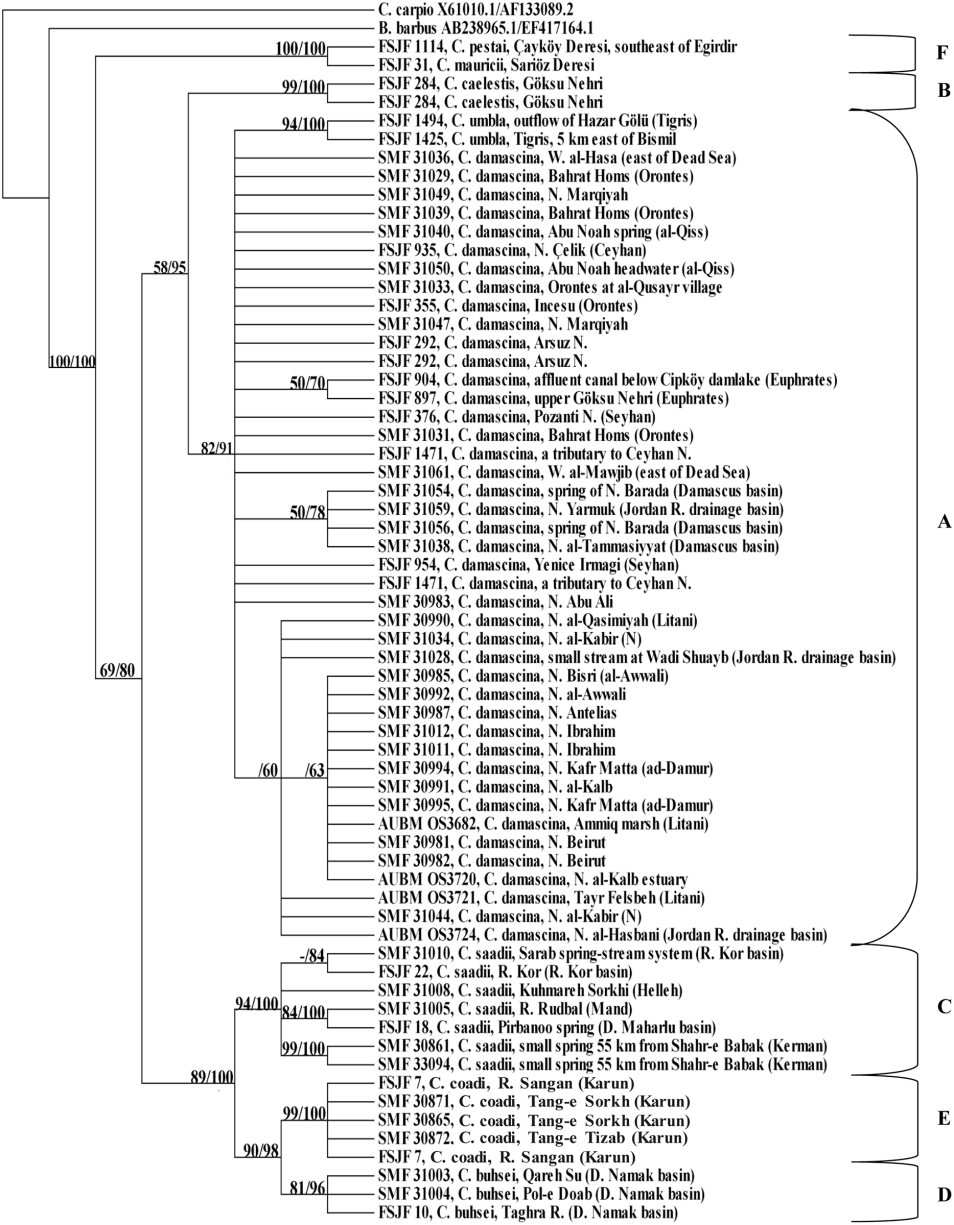
Condensed cladogram obtained from COI+LSU sequences using Maximum Parsimony/Bayesian analysis. Numbers above branches refer to bootstrap/posterior probability percentages; only values ≥ 50% are shown. “-” indicates that no bootstrap value was obtained from MP analysis as only a single sequence was included in the analysis.

The total evidence tree (Fig 6) had a very similar topology to the condensed cladogram obtained from COI sequences, except for very few changes. Although the phylogenetic relationship between *C*. *damascina* and *C*. *umbla* is still not well resolved, specimens of *C*. *umbla* cluster together with each other and form a well-supported monophyletic group (bootstrap value = 94%, PP value = 100%). Similarly, *C*. *buhsei* samples form a well-supported monophyletic group (bootstrap value = 81%, PP value = 96%), which is the sister group to *C*. *coadi*. The phylogenetic relationship between clade F and clade A+B+C+D+E is very well resolved as clade F forms a separate group from clade A+B+C+D+E.

## Discussion

The most important result of the present study is that what was earlier considered *C*. *damascina* in fact represents a complex of six closely related species: *C*. *buhsei* from Daryacheh-ye Namak basin (Iran); *C*. *caelestis* from Göksu Nehri (Turkey); *C*. *coadi* from Rud-e Karun and possibly Rudkhaneh-ye Karkheh; *C*. *damascina* from rivers in the Levant, Mesopotamia and parts of southern Turkey; *C*. *saadii* from rivers draining into the Persian Gulf and the Strait of Hormuz, and from watercourses in the Rud-e Kor, Daryacheh-ye Maharlu, and Kerman basins in Iran; and *C*. *umbla* from the Tigris-Euphrates River system.

Two main lineages were identified within this complex: A western lineage represented by *C*. *caelestis*, *C*. *damascina*, and *C*. *umbla* and an eastern lineage represented by *C*. *buhsei*, *C*. *coadi*, and *C*. *saadii*. This agrees partly with what was published earlier by [38]using the complete cytochrome *b* gene. In their study, the Anatolian species (*C*. *angorae*, *C*. *caelestis*, *C*. *damascina*, and *C*. *kosswigi*) form a sister group to their Iranian congeners (*C*. *buhsei* and *C*. *saadii*). Based on morphological [61] and molecular differences highlighted in our study, *C*. *angorae* is now considered a synonym of *C*. *damascina*. It might well be possible that *C*. *kosswigi* is a member of the *C*. *damascina* species complex but no specimens were available for clarification. According to [38], *Capoeta* specimens from Rud-e Morghab and Rud-e Sangan have been identified as *C*. c.f. *buhsei*. *Capoeta* c.f. *buhsei* from Rud-e Sangan, as shown in our results, and most probably that from Rud-e Morghab, represent a distinct species (*C*. *coadi*). As for the study carried out by [35] on the molecular systematics of the Anatolian *Capoeta* species, we consider his results and conclusions as weak because most of the phylogenetic relationships among the species were not well supported and this led to incorrect conclusions regarding the status of some taxa. For example, he showed that *C*. *kosswigi* and *C*. *umbla* are genetically contiguous and belong to *C*. *trutta*. *Capoeta umbla* proved to be different from *C*. *trutta* and this is very clear based on the results of our study.

The phylogenetic relationships highlighted in our study between *C*. *damascina* and *C*. *umbla* as shown in the condensed cladograms and the sharing of same haplotypes between specimens of *C*. *damascina* from the Euphrates and *C*. *umbla* may be attributed to one of three potential scenarios: The first one is an incomplete lineage sorting due to a very recent speciation; the second one points to a possible mitochondrial transfer in the recent past, where the mitochondrial DNA of *C*. *umbla* was introgressed by *C*. *damascina* from the Tigris-Euphrates River system; and the third one considers a combination of both processes. More ample population sampling of *C*. *damascina* and *C*. *umbla* is needed in order to gain deeper insights into the causative processes. As these two species occur sympatrically in the Tigris-Euphrates River system, it is likely that introgressions would take place as *C*. *damascina* is known to hybridize with species in other genera. For example, a hybrid of *C*. *damascina* and *Luciobarbus longiceps* (Valenciennes in Cuv. and Val., 1842) [18] was described from Lakes Tiberias and Hula by [62]. Hybrids of *C*. *damascina* and *Carasobarbus canis* Valenciennes in Cuv. and Val., 1842 [18] were described and illustrated by [63]from Ain al-Qunaiya, an isolated source within the Jordan River drainage basin.

Regarding the different *C*. *damascina* populations, the relationships among them were not well resolved and no pronounced genetic differences were observed among them. The haplotype network showed that most specimens from the different *C*. *damascina* populations share one of the two most common haplotypes or possess very similar ones. It is important to note that the haplotypes of *C*. *damascina* from the Seyhan Nehri drainage appeared to be more similar to the haplotypes of other *C*. *damascina* populations than to each other. Such results reflect either very recent geographic separation or ongoing gene flow among these populations.

The COI and total evidence trees support the close relationship between *C*. *caelestis* and *C*. *damascina* as well as to *C*. *umbla*, unlike in the tree obtained from LSU sequences, where *C*. *caelestis* formed a separate branch which was basal to all the other clades within *Capoeta*. However, not so much significance should be attached to this as the supports for clade A+C+D+E (bootstrap value = 62%, PP value = 54%) and clade A+C+D+E+F (bootstrap value = 72%, PP value = 61%) were not particularly high. Although linked to clade A in the haplotype network, *C*. *caelestis* forms a separate group (seven steps) and this confirms the results obtained in the phylogenetic trees.

Concerning the eastern lineage, it was shown (based on the COI, total evidence trees, and the haplotype networks) that *C*. *buhsei*, *C*. *coadi*, and *C*. *saadii* were clearly separated from *C*. *damascina*, *C*. *umbla*, and *C*. *caelestis*. This agrees with what has been stated earlier by [59]based on COI and cytochrome *b* sequences. Although the phylogenetic relationships among the clades within the *C*. *damascina* species complex were generally not well resolved using the LSU marker, the tree topology supported the monophyly of *C*. *buhsei*, *C*. *coadi*, and *C*. *saadii*. Interestingly, the *C*. *saadii* haplotypes were quite divergent from the haplotypes of *C*. *buhsei* and *C*. *coadi* (Fig 4) and displayed a pattern without an obvious central haplotype. Thus, it can be concluded that the well-supported mitochondrial lineages of *C*. *saadii* and *C*. *buhsei*/*C*. *coadi* evolved probably under complete genetic isolation. However, the divergence of these evolutionary units was not strong enough to result in a clearly resolved pattern from the less variable ribosomal marker. The split, therefore, most likely occurred rather recently. Contrary to what has been observed in the *C*. *damascina* haplotypes, most of the *C*. *saadii* haplotypes showed differences among the populations. The divergence in mitochondrial sequences among *C*. *saadii* specimens from most of the isolated basins can be interpreted as indication of restricted gene flow among basins. However, with the small number of specimens at hand, it is not possible to assess the significance of the differentiation among putative populations and subpopulations.

**Fig 7:**
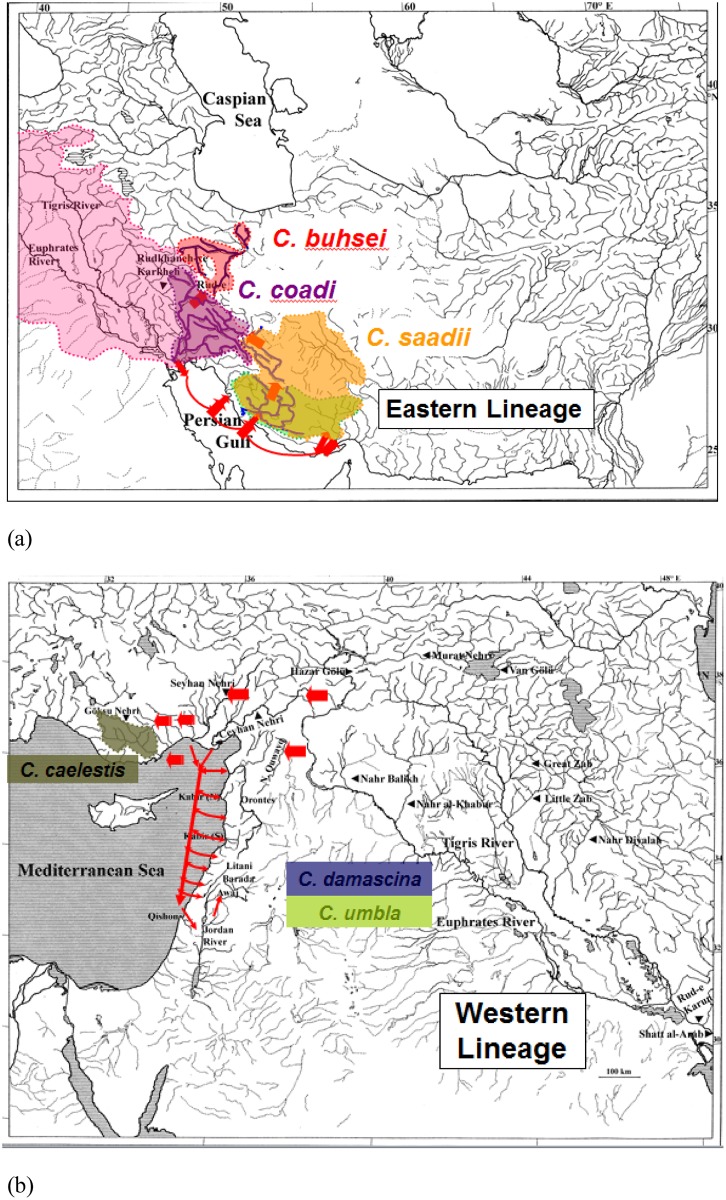
A plausible biogeographical scenario for the separation between the (a) eastern and (b) western lineages.

The results obtained in this study indicate that speciation of members of the *C*. *damascina* species complex is quite recent and that their dispersal and present-day distribution are related to Pleistocene events. During the Pleistocene glacials, when the global sea level dropped by at least 120 m, the Persian Gulf dried up completely and a river valley connected the waters of Mesopotamia to the rivers of the Gulf and Hormuz basins [15,17,64]. It may be assumed that during that period (probably during one of the first glacials), the ancestor of the *C*. *damascina* species complex reached the rivers of the Persian Gulf and Strait of Hormuz basins and differentiated there, giving rise to the eastern lineage which consisted of the ancestor of *C*. *buhsei*, *C*. *coadi*, and *C*. *saadii* (Fig 7a). As the Rud-e Kor basin was part of the Rud-e Mand drainage during that time [65], the ancestor of *C*. *buhsei*, *C*. *coadi*, and *C*. *saadii* most probably reached the Rud-e Kor through this connection (Fig 7a). It possibly reinvaded part of the Tigris-Euphrates River system and from there moved on to the Daryacheh-ye Namak basin through headwater capture during wetter periods of the Pleistocene (Fig 7a). The population in the Gulf, Rud-e Kor, and Hormuz basins then evolved into *C*. *saadii*. It is probable that it made its way into the various basins, where it occurs today (Gulf, Rud-e Kor, Hormuz, Daryacheh-ye Maharlu, and Kerman basins) via headwater capture and/or via more extensive interconnecting watercourses during wet periods of the Pleistocene ([66,67]; Fig 7a). Rivers in these basins have headwaters, which arise in close vicinity of each other on a high plain and transfer of species is expected over time. The sister population from the Iranian Tigris and Namak basins later split into *C*. *coadi* and *C*. *buhsei*.

After the separation from the eastern lineage, the western lineage, which is represented by the ancestor of *C*. *damascina*, *C*. *umbla*, and *C*. *caelestis*, most likely reached the Levant and parts of southern Turkey from the Tigris-Euphrates system during the Pleistocene glacials and after the separation from the eastern lineage (Fig 7b). A connection existed, possibly via headwater capture, in the regions of the upper courses of the Ceyhan Nehri and western affluents to the Euphrates [8]. From the Ceyhan Nehri, it dispersed into the Seyhan Nehri via headwater capture or via the confluence of these two rivers during Pleistocene periods of low sea levels (Fig 7b). It reached the Göksu Nehri following possibly the same routes and evolved into *C*. *caelestis*. The sister population differentiated, most probably in the Tigris-Euphrates River system, into *C*. *damascina* and *C*. *umbla*. Based on the results obtained in this study, it is likely that *C*. *damascina* colonized the Levant and southern Turkey during the Pleistocene glacials. This assumption is supported by the low level of genetic differences among the *C*. *damascina* populations. As connections existed between Tigris-Euphrates and Ceyhan Nehri as well as between Tigris-Euphrates and Nahr Quwayq [4,8], it is very probable that *C*. *damascina* reached Nahr Quwayq and parts of southern Turkey (Ceyhan Nehri) via these routes (Fig 7b). Subsequently, it dispersed from the Ceyhan Nehri to the Seyhan Nehri, as mentioned earlier, either via headwater capture and/or via connections of the lower courses during the Pleistocene periods of low sea levels (Fig 7b). It moved from the rivers of southern Turkey southward to the lower Orontes. These rivers were connected to each other as a result of low sea levels in the eastern Mediterranean [7,8]. The species reached an-Nahr al-Kabir (N) via the confluence of the Ceyhan Nehri and the lower Orontes. It might have colonized the central Orontes, which was represented by the isolated Ghab basin at that time, using two possible routes: Via the Nahr al-Abyad, whose upper reaches were a source of an-Nahr al-Kabir (N) and/or via the coastal rivers in the Nahr Marqiyah area, which were connected to the central Orontes [4,6,8,19]. It got into the upper Orontes via an-Nahr al-Kabir (S), as the former was an upper affluent of the latter [11]. Taking advantage of the low sea levels, it dispersed into the coastal rivers of Syria, Lebanon, and Palestine/Israel (Fig 7b). Another possibility we are considering is that *C*. *damascina* may have dispersed into these rivers via headwater capture or more extensive watersheds during wet periods of the Pleistocene. It colonized the Jordan-Dead Sea drainage basin via the coastal river Nahal Qishon and using the Yizre’el Valley as a pathway (Fig 7b). The flooding of this valley provided swampy connections between the headwaters of Nahal Qishon and streams of Beit She’an in the Jordan Valley [8,12]. During that time, the Damascus basin was still connected to the Jordan River drainage basin [8,10], thus allowing the dispersal of this species into the Damascus basin (Fig 7b).

The low genetic variability among the *C*. *damascina* populations may also be related to the fact that connections between some of the coastal rivers existed until very recently or occasionally still exist allowing for a continuous gene flow between the *C*. *damascina* populations. For example, it is highly possible that Ceyhan and Seyhan were frequently connected as a result of flooding. Today, they are connected by a channel. In addition, part of the water of the Litani River drainage was and is still being diverted to Nahr al-Awwali via Markaba tunnel for the generation of hydroelectric power [68], thus allowing a gene flow between the *C*. *damascina* populations from these two rivers.

As projected above, phylogenetic relationships among members of the *C*. *damascina* species complex reflect the geological history of the area and current patterns of geographic distribution.
